# Return to Golf, Tennis, and Swimming After Elective Cervical Spine Surgery

**DOI:** 10.7759/cureus.9993

**Published:** 2020-08-24

**Authors:** Alexandra Richards, Andrew Pines, Nicolas C Rubel, David Mauler, Joseph Farnsworth, Nan Zhang, Naresh P Patel, Mark Lyons, Matthew Neal

**Affiliations:** 1 Neurosurgery, Mayo Clinic Hospital, Phoenix, USA; 2 Neurosurgery, Mayo Clinic Alix School of Medicine, Scottsdale, USA

**Keywords:** acdf, cervical fusion, cervical laminectomy, return to play, hobby sports, return to sport

## Abstract

Background

After surgery for degenerative cervical spine problems, most patients hope to return to non-competitive sports and other leisure activities. Limited data are available to counsel patients about return to play (RTP) in non-competitive sports after cervical surgery.

Methods

Participants had cervical surgery for degenerative diagnoses from April 1, 2007, to April 1, 2018. Demographic data were collected, and participants were asked to complete a survey regarding sports participation before and after cervical surgery.

Results

Of the 73 participants who responded to the study, the majority (81.1%) were able to return to one or multiple hobby sports after elective spine surgery. RTP rates at 12 months for golf, tennis, and swimming were 67.6%, 31.2%, and 81.6%, respectively. Younger age and lack of preoperative motor deficit were significant predictors of return to swimming after surgery. After surgery, 54.3% of golfers reported similar or improved levels of play.

Conclusions

After elective cervical spine surgery, the majority of hobby athletes can expect to return to athletics. The majority of golfers returned to play with similar or improved frequency and quality of play compared to preoperative levels. Future prospective studies will further elucidate factors predicting RTP after different types of elective cervical surgeries.

## Introduction

Background/rationale

Patients undergo cervical spine surgery to address pain and other neurological symptoms. After surgery for degenerative cervical spine problems, most patients hope to return to non-competitive sports and other leisure activities after surgery. Preoperatively, patients frequently ask whether they will be able to return to sporting activities such as golf, tennis, and swimming. Limited data are available to counsel patients about return to play (RTP) in non-competitive sports after cervical surgery. There are general criteria for return to competitive sport play after spine surgery. There is consensus that postsurgical, competitive athletes, in particular those participating in contact or high-velocity sports, should be pain-free, be neurologically intact, have full strength, and have full range of motion prior to returning to competitive play [1-3].

RTP after surgery for single-level cervical pathology has been previously examined. There is strong consensus in the literature, despite robust evidence-based data, that athletes, including professional athletes, may return to high-velocity and collision sports after single-level anterior cervical discectomy and fusion (ACDF) surgery as well as posterior cervical foraminotomies [4-7]. Reinke et al. also presented a series of 46 athletes ranging from professional level to hobby athlete who all resumed preoperative sporting activity after single-level cervical total disk replacement for soft disk herniation with radiculopathy [8].

Two publications specifically address RTP in golfers after spine surgery. In a case series, Shifflett et al. surveyed patients who underwent one- or two-level lumbar fusion surgery [9]. They found that 52% of patients returned to golf course play within one year from surgery. The majority of golfers returned to preoperative performance levels and frequency of play. In another study, Abla et al. surveyed the North America Spine Society members about suggested timing of return to golf after various spinal surgeries [10]. The most common recommended time for return to golf after lumbar laminectomy and lumbar microdiscectomy was four to eight weeks, after ACDF was two to three months, and after lumbar fusion was six months. There is a paucity of literature available for counseling hobby athletes about RTP, particularly for golf, after cervical surgery for degenerative pathologies.

There are several epidemiological studies demonstrating the high frequency of spine injuries, particularly in junior elite athletes, participating in tennis [3,9] and swimming [2,11]. To the authors’ knowledge, little is known about RTP rates for swimmers and tennis players after cervical surgeries.

This study addresses knowledge gaps for spine surgeons counseling patients about RTP in non-contact hobby sports such as golf, tennis, and swimming. Limited data are available to counsel postoperative spine patients about returning to golf play. To our knowledge, there are no guides for RTP after multilevel anterior cervical fusions or posterior cervical fusions in any of the above-listed sports. In addition, we are unaware of research addressing RTP specifically for swimmers or tennis players after cervical spine surgery.

Objectives

The aim of this study was to determine RTP rates and predictors for RTP for hobby athletes after elective cervical spine surgery. This will aid healthcare providers in counseling patients on RTP after elective cervical surgery.

## Materials and methods

Methods

In this case series study, we first identified patients who underwent cervical surgery for degenerative diagnosis from April 1, 2007, to April 1, 2018, at Mayo Clinic Hospital, Phoenix, AZ. Participants aged 18 to 89 years were included in the study if they underwent an ACDF, posterior cervical fusion, or cervical laminectomy. Exclusion criteria included patients who underwent cervical surgery for non-degenerative pathologies, neurodegenerative disease, simultaneous anterior/posterior cervical surgery, fusions extending into the thoracic spine or fusions extending above cervical 2, revision surgery related to the same surgery, or multi-level corpectomies.

This study had approval from the Institutional Review Board of Mayo Clinic Hospital. Participants consented with a written consent form after being informed about the risks and benefits of study participation.

In our retrospective review, we identified surgery type and patient characteristics that we hypothesized may have impacted RTP. Table 1 includes these details for all surveyed participants who participated in a sport prior to surgery.

**Table 1:** Demographic and Clinical Characteristics ^1^Equal variance two-sample t-test. ^2^Chi-square p-value. ^3^Fisher's exact p-value. ACDF, anterior cervical discectomy and fusion; PCDF, posterior cervical discectomy and fusion

	Participants who participated in any sports before surgery		Total (N=73)	p-Value
	No (N=20)	Yes (N=53)		
Age				0.1038^1^
N (missing)	20 (0)	53 (0)	73 (0)	
Mean (SD)	65.3 (14.1)	69.9 (9.2)	68.7 (10.9)	
Median (IQR)	66 (56-75)	71 (63-76)	69 (62-76)	
Range	33.0-86.0	49.0-90.0	33.0-90.0	
Gender, n (%)				0.1928^2^
Male	12 (60.0%)	40 (75.5%)	52 (71.2%)	
Female	8 (40.0%)	13 (24.5%)	21 (28.8%)	
Are you currently working? n (%)				0.1464^2^
Missing	2	3	5	
No	10 (55.6%)	37 (74.0%)	47 (69.1%)	
Yes	8 (44.4%)	13 (26.0%)	21 (30.9%)	
Have you had more than one cervical spine surgery? n (%)				0.6730^2^
Missing	1	5	6	
No	15 (78.9%)	40 (83.3%)	55 (82.1%)	
Yes	4 (21.1%)	8 (16.7%)	12 (17.9%)	
Surgery type, n (%)				0.8674^3^
ACDF	13 (65.0%)	33 (62.3%)	46 (63.0%)	
Laminectomy	3 (15.0%)	11 (20.8%)	14 (19.2%)	
PCDF	4 (20.0%)	9 (17.0%)	13 (17.8%)	
Other spine surgery, n (%)				0.5084^2^
Missing	1	0	1	
No	11 (57.9%)	26 (49.1%)	37 (51.4%)	
Yes	8 (42.1%)	27 (50.9%)	35 (48.6%)	
Diagnosis of myelopathy, n (%)				0.7432^2^
No	8 (40.0%)	19 (35.8%)	27 (37.0%)	
Yes	12 (60.0%)	34 (64.2%)	46 (63.0%)	
Preoperative sensory deficit, n (%)				0.5506^2^
No	11 (55.0%)	25 (47.2%)	36 (49.3%)	
Yes	9 (45.0%)	28 (52.8%)	37 (50.7%)	
Preoperative motor deficit, n (%)				0.2232^2^
No	13 (65.0%)	26 (49.1%)	39 (53.4%)	
Yes	7 (35.0%)	27 (50.9%)	34 (46.6%)	
Smoker, n (%)				0.5506^2^
No	9 (45.0%)	28 (52.8%)	37 (50.7%)	
Yes	11 (55.0%)	25 (47.2%)	36 (49.3%)	
Diabetic, n (%)				0.5724^2^
No	18 (90.0%)	45 (84.9%)	63 (86.3%)	
Yes	2 (10.0%)	8 (15.1%)	10 (13.7%)	
Cardiac disease/dysfunction, n (%)				0.4711^2^
No	17 (85.0%)	41 (77.4%)	58 (79.5%)	
Yes	3 (15.0%)	12 (22.6%)	15 (20.5%)	

Eligible participants were mailed a survey asking about preoperative participation in the hobby sports of golf, tennis, and swimming. They were also asked about their ability to participate in these activities following their surgery. One month after mailing the envelopes, participants that did not respond were identified. Participants who did not respond received a second mailing with the survey. We did not attempt to contact participants who did not respond after the second survey attempt.

Statistical analysis

Patients were dichotomized into returning to any of the sports or not based on their survey response. Two sample t-test, Wilcoxon rank-sum test, or Fisher’s exact test was used to compare the demographics and clinical characteristics between patients who returned to any of the sports and patients who did not return to identify potential factors that associated with returning to sports.

## Results

We identified 616 cervical procedures performed at our institution from April 4, 2007, April 1, 2018. Of these, 497 patients met our inclusion criteria. We gathered 73 responses to our survey (response rate: 14.7%). The median age of respondents was 69 years, ranging from 33 to 90 years. Of our respondents, 52 (71.2%) were males and 21 (28.8%) were females. In terms of procedures performed, 46 (63%) underwent an ACDF, 14 (19.2%) underwent a cervical laminectomy, and 13 (17.8%) underwent a posterior cervical discectomy and fusion. Of the responding patients, 37 (50.7%) had a preoperative sensory deficit and 34 (46.6%) had a preoperative motor deficit.

Of the respondents, 53 (72.6%) played at least one of the three sports on our survey. Overall, 43 (81.1%) out of 53 of participants who participated in sports before surgery were able to return to their baseline ability. When analyzing the ability of participants to return to playing any of the sports on our survey, we found no significant determining predictors.

Specific to golf, 34 (46.6%) of responding patients played before surgery. Of these, 23 (67.6%) were able to return to play. We found no significant determining predictors of this outcome.

Specific to tennis, 16 (21.9%) of responding patients played before surgery. Of these, 5 (31.2%) were able to return to play. We found no significant determining predictors of this outcome.

Specific to swimming, 38 (52.1%) of responding patients swam before surgery. Of these, 31 (81.6%) were able to return to swimming. We found that both younger age (mean age of 68.2 for those who returned and 77.7 for those who did not return; p=0.0109) and the absence of a preoperative motor deficit (100% patients without preoperative motor deficit returned to swimming, whereas only 69.6% patients with preoperative motor deficit returned to swimming; p=0.0291) were significant determining factors of whether a patient would be able to return to swimming. Table 2 includes descriptive statistics for hobby sports for participants who played hobby sports prior to surgery.

**Table 2:** Descriptive Statistics for Hobby Sports for Participants Who Played Hobby Sports Prior to Surgery

Tennis	Total (N=17)	Swimming	Total (N=40)	Golf	Total (N=35)
Did your spinal problems decrease the frequency of playing tennis? n (%)		Did your spinal problems decrease the frequency of swimming? n (%)		Did your spinal problems decrease the frequency of playing golf? n (%)	
Missing	2	Missing	3	No	10 (28.6%)
No	4 (26.7%)	No	20 (54.1%)	Yes	25 (71.4%)
Yes	11 (73.3%)	Yes	17 (45.9%)		
Was your inability to play tennis a contributing reason for surgery? n (%)		Was your inability to swim a contributing reason for surgery? n (%)		Was your inability to play golf a contributing reason for surgery? n (%)	
Missing	2	Missing	5	Missing	2
No	10 (66.7%)	No	26 (74.3%)	No	18 (54.5%)
Yes	5 (33.3%)	Yes	9 (25.7%)	Yes	15 (45.5%)
Frequency of playing tennis after surgery, n (%)		Frequency of swimming after surgery, n (%)		Frequency of playing golf after surgery, n (%)	
Missing	8	Missing	4	Missing	7
More	3 (33.3%)	More	6 (16.7%)	More	5 (17.9%)
Less	4 (44.4%)	Less	14 (38.9%)	Less	12 (42.9%)
Same	2 (22.2%)	Same	16 (44.4%)	Same	11 (39.3%)
Your level of swimming after surgery, n (%)		Your level of swimming after surgery, n (%)		Your level of playing golf after surgery, n (%)	
Missing	3	Missing	4	Missing	6
Higher	3 (21.4%)	Higher	8 (22.2%)	Higher	7 (24.1%)
Lower	6 (42.9%)	Lower	11 (30.6%)	Lower	10 (34.5%)
Same	5 (35.7%)	Same	17 (47.2%)	Same	12 (41.4%)
When did you resume playing tennis after surgery? n (%)		When did you resume swimming after surgery? n (%)		When did you resume playing golf after surgery? n (%)	
Missing	10	Missing	10	Missing	10
3-6 months	2 (28.6%)	<3 months	10 (33.3%)	<3 months	6 (24.0%)
6-12 months	2 (28.6%)	3-6 months	9 (30.0%)	3-6 months	5 (20.0%)
>12 months	3 (42.9%)	6-12 months	8 (26.7%)	6-12 months	4 (16.0%)
				>12 months	10 (40.0%)

## Discussion

The median age of Americans is increasing [12]. Consequently, healthcare providers will be caring for a growing population of older patients needing cervical surgery for degenerative spine disorders. Many of these patients will participate in sports, as sport participation is one of the most common leisure time physical activities among Americans [3,13]. Older adults are motivated to play sports in order to maintain good health, fitness, and joint mobility. Swimming, golf, and tennis are three of the more common sporting activities reported by older adults [14]. The participants in this study represented an older, active patient population with a median age of 69 years and sport participation rate of 72.6% for swimming, golf, or tennis.

In this cohort, the majority (81.1%) of athletes who participated in a sport including golf, tennis, or swimming before surgery were able to return to a sport after cervical spine surgery. Of the three sports, swimming and golf had the greatest RTP rates at 81.6% and 67.6%, respectively. The mean age for tennis players that returned to sport was 63.2 years. This was the lowest mean age compared to the other two sports. We suspect that both of these findings reflect that tennis play requires more dynamic spine movement and impact compared to the other two sports. These characteristics of tennis may favor younger participants and prevent some postoperative patients from returning to play.

Of the three sports, swimming had the greatest percentage of female participants at 21.1%. This is still a small percentage compared to the percentage of male participants. A recent study in England compared male and female participation in hobby sports including swimming. Similar to the findings in this study, males in the English study had a higher rate of sport participation at 41.2% versus 33.9% for females [15]. Nonetheless, swimming can be an excellent form of exercise for men and women at all stages of life [1].

Among golfers, cervical pathology prior to surgery did decrease the frequency of sport participation in the majority (71.4%) of players. The majority of study participants (57.5%) were able to play after surgery. The players who returned were able to play at the same (39.3%) or a greater (17.9%) frequency after surgery. The players who returned were also able to play at the same (41.4%) or and improved (24.1%) level of play. Similar findings were noted in the only other known publication examining return to golf after spine surgery. Shifflett et al. reported that 52% of golfers were able to return to course play within 12 months after a one- or two-level lumbar fusion for degenerative pathologies [9]. In the same study, the authors also found that 80% of golfers were able to play at the similar or improved handicap. Following elective spine surgery for degenerative cervical and lumbar pathologies, golfers can be expected to return to sport at a high rate with comparable or improved levels of play.

There were several limitations to this study. The cohort size was not large enough for a meaningful analysis of each type of cervical procedure. Therefore, we did not analyze RTP rates for the different cervical procedures. The surveys were performed retrospectively and were subject to recall bias. The survey response rate was only 14.7%, which may be due to outdated contact information as well as using mail for survey collection. If a significant number of the non-responders were able to return to play after surgery, this could significantly change the statistical analysis. Additionally, many of our survey participants identified other hobby sports not initially considered in this study; therefore, future work is needed to examine a greater breadth of hobby sports.

## Conclusions

In this cohort, the majority of hobby athletes were able to return to athletics after elective cervical spine surgery. Among golfers, the frequency and quality of play remained stable or improved postoperatively for the majority of players. Future prospective studies will further elucidate factors predicting RTP after different types of elective cervical surgeries.
